# Supplementary material for: A Global Screen for Assembly State Changes of the Mitotic Proteome by SEC-SWATH-MS
Source: Cell Syst. 2020 Feb 26;10(2):133–155.e6. doi: 10.1016/j.cels.2020.01.001 (PMC7042714; doi:10.1016/j.cels.2020.01.001)

Q9Y262 | EIF3L_HUMAN | EIF3L EIF3EIP EIF3S6IP HSPC021 HSPC025 MSTP005
Monomer MW [kDa]: 66.727 Monomer expected elution fraction: 44

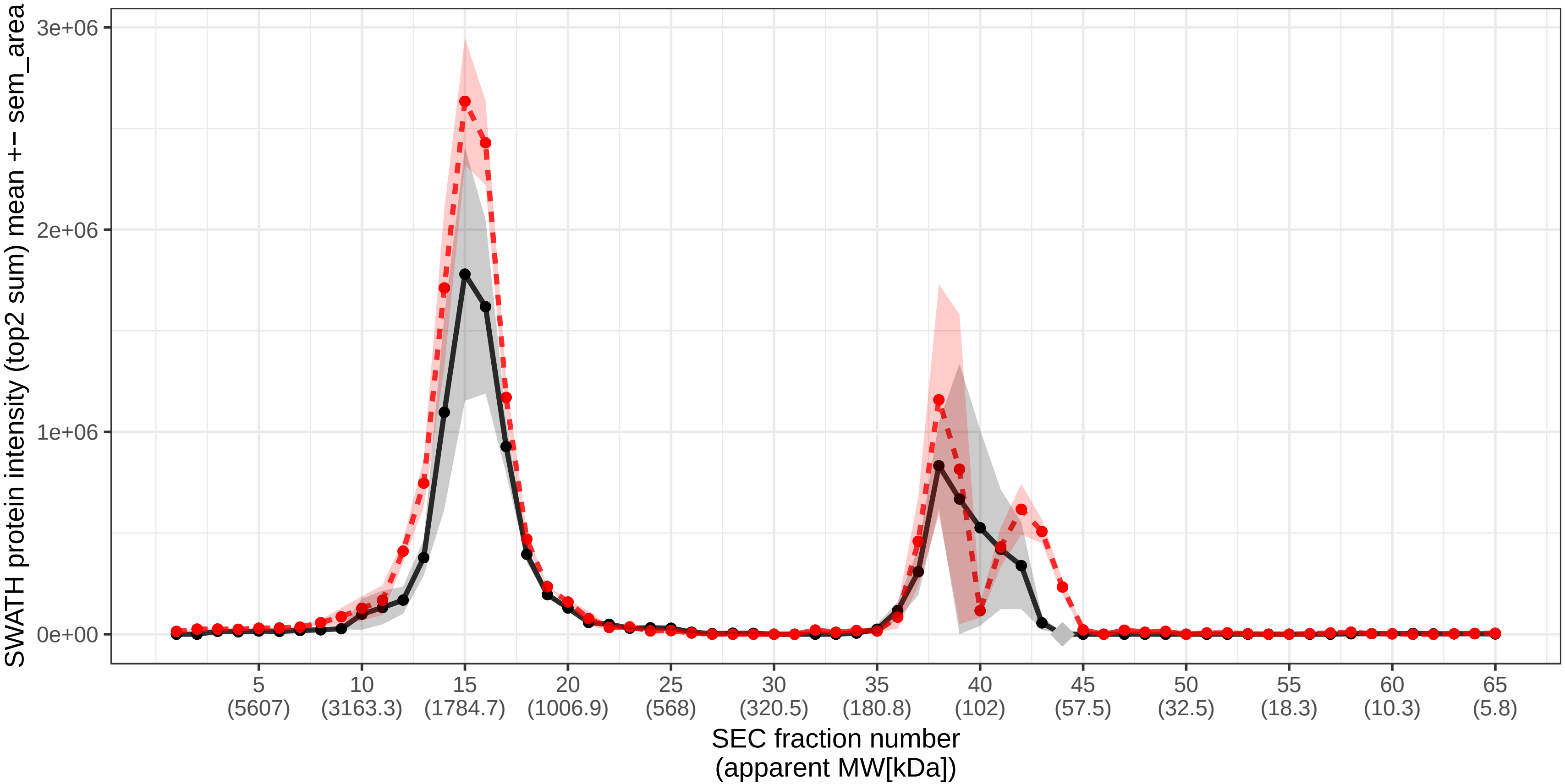

Supplement: Data S1. SEC-SWATH-MS Protein Chromatograms, Related to Figure 1 [file mmc6.zip › SECchrom_Q9Y262_EIF3L_HUMAN_EIF3L_EIF3EIP_EIF3S6IP_HSPC021_.pdf]
